# Supplementary material for: Altered proteome of a Burkholderia pseudomallei mutant defective in short-chain dehydrogenase affects cell adhesion, biofilm formation and heat stress tolerance
Source: PeerJ. 2020 Mar 19;8:e8659. doi: 10.7717/peerj.8659 (PMC7085900; doi:10.7717/peerj.8659)
Supplement: Supplemental Information 6 [file peerj-08-8659-s006.docx]

**Growth kinetics of *B. pseudomallei***

The growth kinetics of *B. pseudomallei* K96243, the SDO mutant, and the SDO complement in LB broth were determined by measuring the optical density at wave length 600 nm. The data points represent mean from triplicate experiments.
